# Are the different cut-off points for sitting time associated with excess weight in adults? A population based study in Latin America

**DOI:** 10.1186/s12889-023-15029-8

**Published:** 2023-01-16

**Authors:** Eduardo Rossato de Victo, Irina Kovalskys, Mauro Fisberg, Georgina Gómez, Attilio Rigotti, Lilia Yadira Cortés, Martha Yépez García, Rossina G. Pareja, Marianella Herrera-Cuenca, Dirceu Solé, Clemens Drenowatz, Adilson Marques, Gerson Ferrari

**Affiliations:** 1 Disciplina de Alergia, Imunologia Clínica e Reumatologia do Departamento de Pediatria da Universidade Federal de São Paulo, São Paulo, Brazil; 2 Carrera de Nutrición, Facultad de Ciencias Médicas, Pontificia Universidad Católica Argentina, Buenos Aires, Argentina; 3 Centro de Excelência em Nutrição e Dificuldades Alimentares (CENDA), Instituto Pensi, Fundação José Luiz Egydio Setubal, Hospital Infantil Sabará, São Paulo, Brazil; 4 Departamento de Pediatria da Universidade Federal de São Paulo, São Paulo, Brazil; 5 Departamento de Bioquímica, Escuela de Medicina, Universidad de Costa Rica, San José, Costa Rica; 6 Centro de Nutrición Molecular y Enfermedades Crónicas, Departamento de Nutrición, Diabetes y Metabolismo, Escuela de Medicina, Pontificia Universidad Católica, Santiago, Chile; 7 Departamento de Nutrición y Bioquímica, Pontificia Universidad Javeriana, Bogotá, Colombia; 8 Colégio de Ciencias de la Salud, Universidad San Francisco de Quito, Quito, Ecuador; 9 Instituto de Investigación Nutricional, La Molina, Lima, Peru; 10 Centro de Estudios del Desarrollo, Universidad Central de Venezuela (CENDES-UCV)/Fundación Bengoa, Caracas, Venezuela; 11 Division of Sport, Physical Activity and Health, University of Education Upper Austria, 4020 Linz, Austria; 12 CIPER, Faculty of Human Kinetics, University of Lisbon, Lisbon, Portugal; 13 ISAMB, University of Lisbon, Lisbon, Portugal; 14 Universidad de Santiago de Chile (USACH), Escuela de Ciencias de la Actividad Física, el Deporte y la Salud, Santiago, Chile; 15 Facultad de Ciencias de la Salud, Universidad Autónoma de Chile, Av. Pedro de Valdivia 425, Providencia, Santiago, Chile

**Keywords:** Sitting time, Sedentary behavior, Obesity, Epidemiologic studies

## Abstract

**Background:**

Excess weight is increasing worldwide, and in Latin America more than half of the population is excess weight. One of the reasons for this increase has been excessive sitting time. Still, it remains to be seen whether there is an excessive amount of that time in Latin American adults. This study aimed to associate different sitting time cut-off points with the excess weight.

**Methods:**

Data from the Latin American Study of Nutrition and Health (ELANS), a cross-sectional population-based survey conducted in eight Latin American countries, were used. The excess weight indicators used were body mass index, and waist and neck circumferences. Sitting time was obtained using questionnaires and categorized at different cut-off points. Differences between sitting time categories (< 4 or ≥ 4; < 6 or ≥ 6; and < 8 or ≥ 8 hours/day) and excess weight were obtained by Student’s t test for independent samples and the association between sitting time categories and different indicators of excess weight were obtained by logistic regression.

**Results:**

The median of the sitting time was 420 min/day (IQR: 240–600). There were no significant differences between body mass index (kg/m^2^) and waist circumference (cm) with categories of sitting time. The mean values of neck circumference (cm) were significantly higher in ≥4, ≥6 and ≥ 8 hours/day than < 4, < 6, and < 8 hours/day of sitting time in the pooled sample. Some distinct differences by country were observed. There were significant differences among excess weight by body mass index (63.2% versus 60.8) with < 8 vs ≥8 hours/day of sitting time. The proportion of excess weight by neck circumference was higher in participants who reported ≥4, ≥6, and ≥ 8 hours/day compared to < 4, < 6, and < 8 hours/day of sitting time. Considering ≥8 hours/day of sitting time, higher odds of excess weight were found evaluated by body mass index (OR: 1.10; 95% CI: 1.01, 1.20) and neck circumference (OR: 1.13; CI 95%: 1.03, 1.24) overall.

**Conclusions:**

Sitting time above 8 hours/day was associated with higher odds of excess weight, even though there were no differences in waist circumference between sitting time categories.

**Trial registration:**

Clinical Trials NCT02226627. (27/08/2014).

## Background

Epidemiological studies have identified that excess weight increases the risk for cardiovascular disease, hypertension, diabetes, and sleep apnea syndrome [1, 2]. The prevalence of excess weight has increased worldwide [1]. Currently, approximately 39% of the adults in the world are excess weight [3]. In Latin America, up to 60% of excess weight has been reported [4]. The population of Latin America is increasingly excess weight. It is estimated that by 2030 more than 80% of the adult population will be excess weight [1, 2].

There are several methods to assess excess weight. Body mass index (BMI) and waist circumference (WC) are the most known and used among them [5]. Although most studies use BMI, there is strong evidence showing that this method has high specificity but low sensitivity for identifying adiposity, as it does not identify half of the people with excess body fat [5, 6]. Recently, neck circumference (NC) has emerged as a simple, inexpensive and convenient screening measure to assess excess weight and also for demonstrating equal or better associations of cardio metabolic risk compared to other standard anthropometric measures such as BMI and WC [7, 8]. Due to the associations observed with several cardiovascular risk factors, neck fat may be a unique and pathogenic deposit of the upper body that could be a measure similar to visceral fat, which has a stronger association with cardio metabolic risks than fat subcutaneous [8, 9].

Concomitantly, sedentary behavior has also been studied and pointed out as an adverse health factor, especially in excess weight [10, 11]. Sedentary behavior has been defined as any waking behavior characterized by an energy expenditure ≤1.5 metabolic equivalents of task (METs), while in a sitting, reclined or lying or reclining posture [12]. Based on self-reports, Latin American adults spent on average 460 min/day sitting and the proportion who reported ≥4, ≥6, and ≥ 8 hours/day was 41, 19 and 7%, respectively [13, 14]. A study carried out with a representative sample in the United States (USA) showed a positive association of self-reported sitting time (ST) with several risk factors, such as WC, BMI, triglycerides, cholesterol (HDL) and insulin [10]. Bullock et al. observed that ≥8 hours of ST a day increased the excess weight (defined as ≥25 kg/m^2^ of BMI) by 62% compared to the reference group (< 4 hours/day), regardless of physical activity, gender and age in 5338 adults from high-income countries (i.e., Europe and United States). The authors found no significant association between other categories of ST (4- < 6 and 6- < 8 hours/day) with obesity compared to the < 4 hours/day [11]. Recent studies indicate that the time in ST is a factor for excess weight and other chronic conditions, regardless the level of physical activity [11, 15].

There are relatively few studies that examined the relationship between different cut-off points of ST with indicators of excess weight (i.e., BMI, WC, and NC) in a large international Latin American sample because of the variety of methodologies applied [16, 17]. Only one of these Latin American studies used a representative sample of the urban population. Paz-Krumdiek et al., found that Peruvian adults with greater ST, using various cut-off points, were more likely to be obese. This association was evident with three different excess weight indicators (i.e., BMI, WC, and waist to height ratio) [16]. The aim of this study was to verify the association between different cut-points of ST with excess weight, using distinct indicators adjusted for physical activity, in a large adult sample from eight Latin American countries.

## Methods

### Study design and sample

The Latin American Study of Nutrition and Health / Estudio Latinoamericano de Nutrición y Salud (ELANS) is a household-based multi-national cross-sectional survey of nationally representative samples from urban populations. The ELANS involved eight Latin American countries (i.e., Argentina, Brazil, Chile, Colombia, Costa Rica, Ecuador, Peru, and Venezuela) stratified by geographical location (only urban areas), gender, age, and socioeconomic status. The ELANS was conducted over 1 year (September 2014 to February 2015). The overarching ELANS protocol was approved by the Western Institutional Review Board (#20140605) and is registered at Clinical Trials (#NCT02226627). All aspects of the study were in accordance with the Declaration of Helsinki. The ethical review boards also approved each site-specific protocol of the participating institutions, and participants` informed consent/assent was obtained. The rationale and design of the study are reported in more detail elsewhere [18, 19].

The sampling size was calculated with a confidence level of 95% and a maximum error of 3.49%. A survey design effect of 1.75 was estimated based on guidance from the U.S. National Center for Health Statistics [18], and calculations of the minimum sample sizes required per socioeconomic level (low, middle, and high), age (15–19.9, 20–34.9, 35–49.9; and 50–65.9 years) and sex (men and women) were performed for each country, resulting in a required sample size of 9090. The study consisted of 9218 (4409 men) participants aged 15–65 years who were chosen using a random complex, multistage sampling frame with a random selection of Primary Sampling Units (PSUs) and Secondary Sampling Units (SSUs). The participants were recruited from PSUs within each selected city in each country. An “n” size proportional to population weight was used to select PSUs. In this instance, a simple random sampling of “n” with replacement was achieved to adhere to the principle of statistical independence of selecting the areas included in the PSU sample. For these random selections, the probability proportional to size method was applied. Thus, within each area included in the PSU distribution, a representative sample of SSUs was randomly designated using the probability proportional to size method. Households within each SSUs were selected based on systematic randomization. Details about participant sampling and recruitment strategies have been published elsewhere [18].

A total of 9218 (47.8% men) participants (aged 15.0–65.0 years) were included in the ELANS study. In the present study, data on adolescents aged between 15 and 19 were excluded because of the different cut-offs used for excess weight in adolescents and adults [20–22]. Therefore, the present study included a final sample of 7995 (53.4% women), aged between 20 and 65 years.

### Sitting time

ST was evaluated based on self-reported time in a sitting position, using a Spanish and Portuguese language long-form “last seven days” of the International Physical Activity Questionnaire (IPAQ), which has been validated in both languages for the assessment of physical activity and ST [23–26].

Participants were asked to estimate the amount of time (min/day) spent sitting at work, at home, and during leisure time [27]. (I) “How many days did you use the computer at home in the last 7 days?” (II) “During the last 7 days, did you remain sitting?” (Yes, No); (III) “During the last 7 days, on how many days did you remain sitting?” (IV) “How much time did you usually remain sitting for?” These questions were asked separately for weekdays and weekend days. We summed weekday and weekend day ST to calculate average daily overall ST as follows (weekday time*5 + weekend day time*2)/7.

We adopted three cut-off points (≥4, ≥6, and ≥ 8 hours/day) for ST, which have been recognized as critical points for several negative outcomes, including risk of cardiovascular disease and all-cause mortality and have been widely used in previous research [28, 29]. Due to inconsistency concerning cut-off points across health outcomes, there currently is lack research on which to base specific public health recommendations (a single cut-off) regarding the appropriate limit of sedentary behavior to maximize cardiovascular disease health benefits [30].

### Excess weight

Excess weight measurements were taken by previously trained professionals using standard protocols. Bodyweight was measured to the nearest 0.1 kg using a portable scale (Seca®, model, Hamburg, Germany), which had an upper limit of 200 kg. Heavy clothing, pocket items, shoes, and socks were removed [31]. Two measurements were obtained, and the average was used for analysis (a third measurement was obtained if the first two measurements were > 100 g apart, and the closest two measurements were averaged for analysis).

The participants’ body height was measured using a portable Seca 213® stadiometer (Hamburg, Germany), whose measuring range was from 0 to 205 cm. An individual’s height was measured without shoes (either barefoot or with socks) [31]. The individual was positioned under the stadiometer, standing in an erect position with the back against the wall, and remained with feet together, heel against the wall, heels, buttocks, back and head touching the wall, knees straight, and looking forward with head positioned in the Frankfurt plane. The measurement was taken during inspiration and was repeated with the average of both measurements being used for analysis (a third measurement was obtained if the first two measurements were > 0.2 cm apart, and the average of the two closest measurements was used for analysis). BMI (kg/m^2^) was calculated based on reference data for adults [32, 33] BMI was categorized into two categories (eutrophic vs excess weight). Values less than 25 were classified as eutrophic. Values greater than or equal to 25 were classified as excess weight [33].

WC was measured to the nearest centimeter, midway between the lowest rib and the iliac crest after a regular expiration, with the subject standing, according to World Health Organization (WHO) recommendations [32, 33]. The WC measurement was conducted on the skin using an inelastic tape after removing any accessories, such as belts and girdles, situated in the abdominal area. The individual was standing with feet together and arms positioned beside the body. WC measurement was performed midway between the lowest rib and the iliac crest after normal breath. The measurement was repeated, and the average was used for analysis (a third measurement was obtained if the first two measurements were > 1 cm apart and the average of the two closest measurements was used for analysis). WC was categorized based on reference data for adults and sex [34]. In women, values equal to or less than 88 cm were classified as eutrophic and values greater than 88 were classified as excess weight. In men, those with a value equal to or smaller than 102 cm were considered eutrophic, and higher values were classified as excess weight [34].

NC (in centimeters) was measured at the point just below the larynx (thyroid cartilage) and perpendicular to the long axis of the neck (with the tape line in the front of the neck at the same height as the tape line at the back of the neck) using an inelastic tape measure [35]. Each measurement was repeated twice to ensure accuracy, and the average was used for the analyses. A third measurement was taken if the two readings differed by more than the previously established setpoint (0.5 cm for NC). All three measurements were recorded, and the closest two measurements were used to calculate the average. Women with NC > 35 and men with > 39 were classified as excess weight. Those with equal or lower values were considered eutrophic [36].

### Correlates

Age, sex, marital status, work status, race/ethnicity, socioeconomic level, physical activity, and energy intake were assessed using standard questionnaires that were completed during face-to-face interviews and included as covariates in all statistical models. Marital status was classified as married or not-married (single, widowed or divorced). Work status was categorized as working (half-time and full-time) or not-working (student, unemployed, retired, and others). Race/ethnicity was classified as White/Caucasian, Black, Mixed (born of father and mother of different Race/Ethnicities), or Others (Asian, Indigenous, and others). Socioeconomic level data was divided into three strata (low, medium, and high) based on the national indexes used in each country. Further details on sociodemographic characteristics by countries can be found elsewhere [13, 19, 37, 38].

Physical activity was evaluated using the International Physical Activity Questionnaire (IPAQ). Participants were instructed to report the frequency and duration (bouts of > 10 minutes) of physical activity in active transport and leisure-time [27]. IPAQ physical activity data are reported as min/week of walking, moderate and vigorous physical activity during leisure-time, and min/week of walking and cycling for transport-related purposes. Time (min/week) spent in each physical activity domain (i.e., transport-related and leisure-time) was calculated. The sum of active transport (walking + bicycle) and leisure-time physical activity (walking + moderate + vigorous) was considered total physical activity [19, 27]. Participants were categorized as active (≥150 minutes/week) or insufficiently active (< 150 minutes/week) moderate-to-vigorous physical activity guidelines [39].

Dietary intake data was obtained from two in-person 24 h dietary recall interviews using the automated multiple-pass method [40]. Foods and beverages were converted into energy and nutrients using the Nutrition Data System for Research Software (NDS-R version 2013) [41]. Energy intake was also used as potential confounder to evaluate the associations between ST and excess weight indices. Detailed information can be found in a previous publication [18, 42].

### Statistical analyses

According to sociodemographic characteristics, sex, socioeconomic level, and country, weighting was done [19]. A Kolmogorov-Smirnov test was applied to evaluate the data distribution and the Levene test for analysis of homogeneity of variances. Descriptive statistics included means, standard deviations (SD), or frequencies as appropriate. Due non normal of ST, data is presented as median and interquartile range (IQR: 25th and 75th) values. Differences between ST categories (< 4 or ≥ 4; < 6 or ≥ 6; and < 8 or ≥ 8 hours/day) and excess weight (BMI, WC, and NC) were analyzed using a Student’s t test for independent samples for continuous data, and chi-square test were used for categorical data.

Logistic regression models examined the association between each ST category (independent variable) with BMI, WC and NC (dependent variable). Our model was adjusted for sex, age, marital status, race/ethnicity, socioeconomic level, physical activity, and energy intake. All statistical analyses were performed using SPSS V22 software (SPSS Inc., IBM Corp., Armonk, New York, NY, USA). We present the overall (i.e., pooled) and country-specific results. The level of significance was set at *p* < .05.

## Results

The sample consisted of 7995 adults with a mean age of 38.7 years (SD: 12.8). Overall, 52.8% of the sample consisted of female, 53.8% were married, 59.2% were working, 45.7% were classified as being of Mixed race/ethnicity, and 51.9%% had low socioeconomic level. A total of 50.0% of participants not met the moderate-to-vigorous physical activity guidelines. The mean of energy intake was 1993.3 (SD: 620.3) kcal/day. The country with the highest proportion of participants was Brazil (22.1%) and the country with the lowest proportion was Ecuador 8.4%. The median of the ST was 420 min/day (IQR: 240–600), being lowest in Ecuador (300 min/day [IQR: 180–480]) and highest in Argentina (480 min/day [IQR: 330–720]) and Peru (480 min/day [IQR: 315–660]), respectively. In relation to excess weight indicators, the entire sample had a mean of 27.2 kg/m^2^ (SD: 5.5), 89.2 cm (SD: 14.0) and 35.7 cm (SD: 4.0) of BMI, WC and NC (Table 1).

**Table 1:** Descriptive analysis (mean [SD]) of age, sitting time, body mass index, waist and neck circumference of adults by country

Variables	N	Age	Sitting time (min/day)^a^	Body mass index (kg/m^2^)	Waist circumference (cm)	Neck circumference (cm)
All sample	7995	38.7 (12.8)	420 (240–600)	27.2 (5.5)	89.2 (14.0)	35.7 (4.0)
Sex						
Men	3729	37.7 (12.7)	420 (240–660)	26.9 (5.1)	91.3 (13.6)	38.1 (3.7)
Women	4266	39.5 (12.8)	389 (240–600)	28.0 (5.8)	88.7 (14.0)	33.9 (3.3)
Marital status						
Married	4307	40.5 (12.0)	375 (240–600)	28.1 (5.3)	91.7 (13.5)	36.1 (4.1)
Not-married	3688	36.5 (13.3)	420 (240–659)	26.8 (5.6)	88.0 (14.1)	35.7 (4.0)
Work status						
Working	4730	38.8 (11.6)	420 (240–620)	27.5 (5.3)	90.4 (36.5)	36.5 (4.1)
Not-working	3265	38.4 (14.4)	390 (240–600)	27.6 (5.8)	89.3 (14.4)	34.9 (3.8)
Race/ethnicity						
White/Caucasian	2794	39.8 (12.8)	420 (240–600)	27.8 (5.7)	90.8 (14.7)	35.9 (4.2)
Black	522	36.6 (12.1)	390 (240–600)	27.4 (5.8)	90.5 (14.4)	35.7 (4.4)
Mixed	3653	38.0 (12.7)	420 (240–600)	27.3 (5.3)	89.4 (13.1)	35.9 (3.9)
Others	1026	38.9 (12.7)	330 (130–600)	27.2 (4.8)	88.9 (12.1)	34.6 (4.6)
Socioeconomic level						
Low	4149	38.2 (13.0)	360 (210–600)	27.5 (5.6)	89.8 (13.9)	35.7 (4.1)
Medium	3073	38.4 (12.6)	420 (240–660)	27.5 (5.3)	90.1 (13.9)	36.1 (4.1)
High	773	38.8 (12.3)	480 (300–660)	27.7 (5.3)	90.4 (4.1)	38.8 (12.3)
Physical activity						
Active	3955	38.0 (12.7)	390 (240–600)	27.2 (5.3)	89.5 (13.4)	35.8 (3.9)
Insufficiently active	4000	39.3 (12.8)	420 (240–660)	27.9 (5.8)	90.7 (14.5)	35.9 (4.2)
Country						
Argentina	1114	39.5 (12.6)	480 (330–720)	27.4 (5.9)	89.5 (15.4)	35.7 (4.0)
Brazil	1765	39.1 (12.6)	360 (210–600)	27.0 (5.6)	88.4 (14.4)	34.9 (4.5)
Chile	761	39.4 (12.8)	420 (300–600)	28.4 (5.4)	93.2 (14.0)	37.4 (3.9)
Colombia	1082	39.6 (13.5)	420 (240–660)	26.0 (5.0)	85.9 (12.8)	35.1 (3.5)
Costa Rica	677	38.4 (12.6)	360 (240–600)	28.0 (6.1)	93.0 (15.1)	36.9 (3.8)
Ecuador	672	37.5 (12.9)	300 (180–480)	27.1 (5.3)	88.4 (11.9)	35.2 (3.7)
Peru	948	37.2 (12.5)	480 (315–660)	27.0 (4.8)	88.3 (12.0)	35.5 (3.5)
Venezuela	976	37.8 (12.7)	360 (180–540)	27.6 (5.7)	89.9 (14.2)	36.3 (4.1)

^a^ as median and interquartile range (25th and 75th) values

Table 2 presents results from BMI (kg/m^2^), WC (cm) and NC (cm) across categories of ST. There were no significant differences among categories of ST for BMI and WC in total sample and by country. On the other hand, the mean values of NC were significantly higher in ≥4, ≥6 and ≥ 8 hours/day than < 4, < 6, and < 8 hours/day of ST in the pooled sample. Some distinct differences by country were observed. Only in Brazil and Colombia presented significant differences between all categories of ST (Table 2).

**Table 2:** Comparison (mean [SD]) between sitting time categories with body mass index, waist and neck circumference of adults by country

	< 4 hours/day	≥4 hours/day	< 6 hours/day	≥6 hours/day	< 8 hours/day	≥8 hours/day
**Body mass index (kg/m**^**2**^**)**						
Argentina	27.69 (5.91)	27.45 (5.91)	27.54 (5.67)	27.47 (6.02)	27.25 (5.53)	27.72 (6.62)
Brazil	26.68 (5.58)	27.20 (5.66)	26.83 (5.54)	27.22 (5.73)	27.02 (5.59)	27.04 (5.72)
Chile	28.70 (5.18)	28.43 (5.46)	28.45 (5.03)	28.50 (5.63)	28.63 (5.43)	28.26 (5.36)
Colombia	26.18 (4.79)	25.97 (5.07)	26.09 (4.77)	25.98 (5.15)	26.00 (4.70)	26.07 (5.34)
Costa Rica	27.62 (5.77)	28.28 (6.27)	27.89 (5.68)	28.24 (6.53)	28.14 (5.91)	27.95 (6.51)
Ecuador	27.15 (5.20)	27.17 (5.41)	27.10 (5.07)	27.26 (5.71)	27.10 (5.12)	27.35 (5.89)
Peru	26.88 (4.66)	27.06 (4.92)	27.03 (4.49)	27.03 (5.05)	26.96 (4.38)	27.10 (5.33)
Venezuela	27.79 (5.86)	27.58 (5.67)	27.70 (5.73)	27.60 (5.73)	27.67 (5.81)	27.61 (5.60)
All sample	27.18 (5.47)	27.31 (5.59)	27.22 (5.35)	27.32 (5.71)	27.27 (5.41)	27.28 (5.76)
**Waist circumference (cm)**						
Argentina	89.84 (14.73)	89.45 (15.56)	90.52 (14.34)	89.05 (15.87)	89.75 (14.00)	89.30 (16.65)
Brazil	87.99 (14.37)	88.66 (14.42)	88.15 (14.03)	88.74 (14.75)	88.42 (14.11)	88.50 (14.90)
Chile	93.60 (13.62)	93.14 (14.11)	92.99 (13.22)	93.39 (14.50)	93.57 (13.98)	92.72 (14.04)
Colombia	86.02 (12.03)	85.86 (13.15)	85.65 (12.19)	86.08 (13.33)	85.70 (12.26)	86.15 (13.58)
Costa Rica	92.35 (14.88)	93.30 (15.25)	92.87 (14.04)	93.13 (16.15)	92.98 (14.25)	93.03 (16.68)
Ecuador	88.84 (11.35)	88.16 (12.40)	88.35 (11.26)	88.63 (13.00)	88.43 (11.45)	88.53 (13.35)
Peru	88.10 (11.42)	88.38 (12.16)	88.59 (11.11)	88.21 (12.47)	88.17 (10.84)	88.51 (13.16)
Venezuela	90.55 (14.05)	89.60 (14.28)	90.09 (13.77)	89.73 (14.62)	89.92 (13.72)	89.88 (15.04)
All sample	89.22 (13.62)	89.28 (14.21)	89.26 (13.35)	89.26 (14.58)	89.32 (13.43)	89.17 (14.91)
**Neck circumference (cm)**						
Argentina	35.26 (4.52)	35.79 (3.89)	35.48 (4.25)	35.80 (3.90)	35.65 (4.11)	35.74 (3.93)
Brazil	34.39 (4.43)	35.17 (4.58)^a^	34.58 (4.44)	35.25 (4.63)^a^	34.71 (4.49)	35.62 (4.63)^a^
Chile	37.42 (3.83)	37.45 (3.93)	37.35 (3.81)	37.50 (3.97)	37.42 (3.87)	37.48 (3.97)
Colombia	34.97 (3.31)	35.51 (3.59)^a^	35.05 (3.37)	35.60 (6.62)^a^	35.10 (3.40)	35.71 (3.66)^a^
Costa Rica	36.43 (3.62)	37.13 (3.96)^a^	36.76 (3.70)	37.07 (4.03)	36.71 (3.69)	37.28 (4.16)^a^
Ecuador	34.91 (3.67)	35.51 (3.71)^a^	35.07 (3.64)	35.53 (3.79)	35.15 (3.68)	35.56 (3.76)
Peru	35.05 (3.30)	35.70 (3.63)^a^	35.49 (3.34)	35.64 (3.70)	35.36 (3.35)	35.83 (3.80)^a^
Venezuela	36.52 (4.19)	36.31 (4.14)	36.22 (3.99)	36.53 (4.30)	36.26 (4.11)	36.60 (4.23)
All sample	35.39 (4.08)	35.93 (4.06)^a^	35.54 (4.02)	35.98 (4.11)^a^	35.63 (4.03)	36.01 (4.13)^a^

^a^Student’s t test for independent samples for comparison (*p* < 0.05) between < 4 and ≥ 4; < 6 and ≥ 6; and < 8 and ≥ 8 hours/day

Table 3 shows the proportion of excess weight participants based on BMI, WC, and NC for each ST category across the entire sample and by country. There were significant differences among excess weight by BMI (63.2% versus 60.8) with < 8 vs ≥8 hours/day of ST in the total sample. In all categories of ST, the proportion of excess weight by NC was higher in participants who reported ≥4, ≥6, and ≥ 8 hours/day compared to < 4, < 6, and < 8 hours/day of ST. However, there were no significant differences for all categories of ST and excess weight by WC (Table 3).

**Table 3:** Proportion (%) of excess weight of body mass index, waist and neck circumference according sitting time categories of adults by country

	< 4 hours/day	≥4 hours/day	< 6 hours/day	≥6 hours/day	< 8 hours/day	≥8 hours/day
**Body mass index – Excess weight (%)**						
Argentina	62.3	61.0	62.6	60.6	61.8	60.7
Brazil	58.7	60.8	60.4	59.9	61.5	57.9
Chile	73.3	71.2	73.3	70.6	73.5	68.8
Colombia	55.8	52.3	53.8	52.8	53.4	52.9
Costa Rica	62.7	66.9	65.7	65.6	66.7	63.6
Ecuador	66.0	62.0	64.2	63.1	63.2	65.4
Peru	62.4	63.8	65.8	62.5	65.6	61.5
Venezuela	65.5	64.4	65.8	63.8	64.4	65.4
All sample	62.3	62.2	63.1	61.5	63.2	60.8^a^
**Waist circumference – Excess weight (%)**						
Argentina	36.8	34.8	35.3	35.1	34.7	35.7
Brazil	32.3	32.0	32.3	31.9	33.2	30.4
Chile	43.0	41.6	41.8	42.0	42.3	41.4
Colombia	25.7	23.2	23.9	23.9	23.4	24.5
Costa Rica	38.6	43.0	39.6	43.6	41.8	41.3
Ecuador	33.0	29.5	31.6	30.1	31.7	29.2
Peru	36.2	31.4	35.6	30.5	32.9	31.5
Venezuela	36.9	36.1	37.3	35.5	35.9	37.2
All sample	34.3	33.4	33.9	33.4	34.0	33.1
**Neck circumference – Excess weight (%)**						
Argentina	33.0	35.0	34.5	34.8	34.8	34.5
Brazil	25.0	29.7^a^	26.0	30.2^a^	26.9	30.4
Chile	53.3	52.8	52.2	53.3	53.2	52.5
Colombia	23.4	27.4	23.8	28.2	24.1	29.1
Costa Rica	41.2	48.0	45.4	46.3	45.4	46.9
Ecuador	26.2	29.8	27.3	29.8	26.8	32.4
Peru	30.5	31.5	30.5	31.7	28.5	34.3^a^
Venezuela	41.1	41.3	40.3	42.1	39.5	44.3
All sample	32.0	35.7^a^	33.2	35.8^a^	33.5	36.4^a^

^a^ Chi-square test (*p* < .05) for comparison between each categories of sitting time (< 4 vs ≥4 hours/day; < 6 vs ≥6 hours/day; < 8 vs ≥8 hours/day) with body mass index, waist circumference and neck circumference (eutrophic vs excess weight)

Table 4 expresses the association between ST categories and excess weight indicators. There were no significant differences in excess weight based on BMI or WC among ≥4 and ≥ 6 horas/day across the total sample and by country. Using NC as an excess weight indicator, there were, however, significantly higher excess weight rates in the > 4 (OR: 1.17; 95%CI: 1.06, 1.30) and ≥ 6 hours/day (OR: 1.12; 95%CI: 1.02, 1.23) of ST across the total sample. These results were only observed for Brazil in the country-specific analyses (≥4 horas/day; OR: 1.26; 95%CI: 1.01, 1.57 and ≥ 6 horas/day; OR: 1.23; 95%CI: 1.01, 1.50). Considering ≥8 hours/day of ST, we found higher odds of excess weight assessed by BMI (OR: 1.10; 95%CI: 1.01, 1.20) and NC (OR: 1.13; 95%CI: 1.03, 1.24) in the total sample. Peru was the only country with higher odds of excess weight measured by NC (OR: 1.13; 95%CI: 1.00, 1.71) (Table 4).

**Table 4:** Logistic regression model between sitting time categories and body mass index, waist and neck circumference of adults in overall by country

	Body mass index (0 = normal weight; 1 = excess weight)	Waist circumference (0 = normal weight; 1 = excess weight)	Neck circumference (0 = normal weight; 1 = excess weight)
	OR (95% CI)	OR (95% CI)	OR (95% CI)
**≥4 horas/day**			
Argentina	0.94 (0.69, 1,29)	1.09 (0.80, 1.48)	1.09 (0.79, 0.49)
Brazil	1.09 (0.89. 1.33)	1.01 (0.82, 1.25)	**1.26 (1.01, 1.57)**
Chile	0.89 (0.61, 1.32)	1.05 (0.74, 1.49)	0.97 (0.69, 1.37)
Colombia	0.86 (0.66, 1.13)	1.14 (0.84, 1.53)	1.23 (0.90, 1.67)
Costa Rica	1.20 (0.86, 1.66)	0.83 (0.60, 1.14)	1.31 (0.95, 1.80)
Ecuador	0.83 (0.61, 1.13)	1.17 (0.86, 1.60)	1.19 (0.86, 1.65)
Peru	1.06 (0.75, 1.49)	1.24 (0.82, 1.74)	1.05 (0.73, 1.49)
Venezuela	0.95 (0.72, 1.25)	1.03 (0.79, 1.35)	1.01 (0.77, 1.31)
All sample	0.99 (0.89, 1.09)	1.04 (0.94, 1.15)	**1.17 (1.06, 1.30)**
**≥6 horas/day**			
Argentina	0.92 (0.71, 1.18)	1.01 (0.77, 1.30)	1.01 (0.78, 1.30)
Brazil	0.97 (0.81, 1.17)	1.01 (0.83, 1.23)	**1.23 (1.01, 1.50)**
Chile	0.87 (0.63, 1.20)	0.99 (0.74, 1.32)	1.04 (0.78, 1.38)
Colombia	0.95 (0.75, 1.21)	0.99 (0.75, 1.31)	1.25 (0.95, 1.64)
Costa Rica	0.99 (0.73, 1.35)	0.84 (0.63, 1.14)	1.03 (0.77, 1.38)
Ecuador	0.95 (0.70, 1.30)	1.07 (0.77, 1.48)	1.13 (0.81, 1.57)
Peru	0.86 (0.66, 1.14)	1.25 (0.95, 1.66)	1.05 (0.79, 1.40)
Venezuela	0.91 (0.71, 1.17)	1.08 (0.84, 1.39)	1.08 (0.84, 1.38)
All sample	0.93 (0.85, 1.02)	1.02 (0.93, 1.12)	**1.12 (1.02, 1.23)**
**≥8 horas/day**			
Argentina	1.05 (0.83, 1.32)	0.95 (0.75, 1.21)	0.98 (0.77, 1.25)
Brazil	1.16 (0.96, 1.40)	1.13 (0.93, 1.39)	1.18 (0.96, 1.45)
Chile	1.25 (0.92, 1.71)	1.03 (0.78, 1.38)	0.97 (0.73, 1.28)
Colombia	1.02 (0.81, 1.29)	0.94 (0.71, 1.23)	1.28 (0.99, 1.67)
Costa Rica	1.14 (0.83, 1.57)	1.01 (0.74, 1.38)	1.06 (0.78, 1.44)
Ecuador	0.90 (0.64, 1.28)	1.12 (0.78, 1.62)	1.31 (0.91, 1.88)
Peru	1.19 (0.92, 1.54)	1.07 (0.82, 1.39)	**1.31 (1.00, 1.71)**
Venezuela	0.95 (0.73, 1.24)	0.94 (0.72, 1.22)	1.22 (0.94, 1.57)
All sample	**1.10 (1.01, 1.20)**	1.04 (0.95, 1.14)	**1.13 (1.03, 1.24)**

Logistic regression model with body mass index, waist and neck circumference as dependent variable and sitting time as independent variable adjusted for age, sex, marital status, work status, race/ethnicity, socioeconomic level, physical activity, and energy intake

OR Odds ratio, 95%CI Confidence interval 95%

## Discussion

The present study aimed to investigate the association between different cut-off points of ST with excess weight, using distinct indicators (i.e., BMI, WC, and NC) in adults from eight Latin American countries. The total sample > 4 and ≥ 6 hours/day were associated with higher odds of excess weight evaluated by NC. Only Brazil, presented higher odds between ≥4 and ≥ 6 hours/day of ST and excess weight evaluated by NC. Considering ≥8 hours/day of ST, we found higher odds of excess weight assessed by BMI and NC in the total sample. Peru was the only country with higher odds of excess weight measured by NC using an 8 hours/day cut-point.

The present study categorized ST using different cut-points (4, 6, and 8 hours/day) from the perspective of finding an “acceptable” value of ST that did not significantly increase the excess weight. There is still no maximum value in the literature that establishes the amount of time sitting where the health risk is lower. The main recommendation on the subject is that we should minimize ST as much as possible, the World Health Organization itself, in its guideline, only reiterates that adults should limit the amount of sedentary behavior and, if possible, replace this time with physical activities of any intensity [43]. Still, several studies have shown the harmful effects of ST on health associated with cardio metabolic risk and obesity biomarkers [44, 45]. Given the gap in the literature on the acceptable amount of ST, studies have commonly used ST quartiles to establish the range of time where the risk is greatest for health or categorizing into low, medium and high ST [44–46]. Even without this cut-off point for ST, this behavior is seen as an independent factor for the excess weight [11, 47].

This study showed that, regardless of the category of ST, the excess weight as seen by NC was always higher in the groups with the highest number of hours spent sitting compared to the group with the lowest amount. Therefore, we can say that NC was more sensitive to the effects of ST, since WC did not present a significant association between the groups. BMI only showed an increased excess weight when comparing the > 8 hours and < 8 hours’ groups. The hypothesis of this study was that ST was associated with excess weight and was significant in the three indicators analyzed, as seen in other studies that correlated ST or sedentary behavior with the main indicators of excess weight [16, 48, 49]. In addition, due to the moderate to strong correlation between these three indicators, it was believed that all three would show similar results [50]. However, the results were not significant for WC, although BMI and NC demonstrate risk related to ST. To justify such findings, we can rely on the limitations of WC measurement already mentioned in other articles, which does not diminish its importance, much making its use less feasible [51]. The cutoff point adopted for this study may have been a possible reason for not finding significant differences. In addition, the categories of ST used in this study were perhaps not sufficient to show significant differences, demonstrating a greater sensitivity of NC to other indicators of excess weight.

It is also worth noting the capacity and function that each indicator has in the context of excess weight and its possible relationships with ST. BMI classifies, through height and weight, into underweight, normal weight, overweight or excess weight and also indicates obesity. However, it does not provide information on the distribution of body fat. WC is an indicator of fat distribution but it can be easily affected by postprandial abdominal distention, respiratory movement and susceptibility to numerous measurement errors [51, 52]. On the other hand, NC does not vary throughout the day, as it does not change after a meal, and has an easier measurement for the evaluator compared to WC, which may explain the stronger association with obesity [53–55]. It should also be considered that WC cut-off values for excess weight vary widely across geographic regions of the world [56]. Perhaps NC does not show such variation between countries and regions, proving to be a more universal indicator for obesity in multinational studies.

According to the ST values of this Latin American sample, adults spend 4 to 10 hours/day in this behavior. A scoping review with data from 62 countries around the world showed that the median population ST was 4.7 hours/day [57]. Even though most of the studies selected for the review used the IPAQ to measure ST, the authors commented that the population of high-income countries tended to report more time sitting when compared to low-income countries, which may explain the difference to the ST reported in the present study. Our study did not consider the socioeconomic level of the country for comparison purposes. Still, in the case of Latin America, where countries are from low to middle income, ST seemed to be quite high if we consider the values presented by the countries in a review [57].

Two countries in our study (Brazil and Peru) showed a significant difference between sitting time and excess weight. For instance, the mean values of NC were significantly higher in ≥4, ≥6 and ≥ 8 hours/day than < 4, < 6, and < 8 hours/day of ST in Brazil. Sociodemographic variances between inhabitants living in the Latin America can also help clarify ST and excess weight differences [24, 26]. Presumably people with higher education and socioeconomic status have more sedentary jobs, are more likely to use cars than active transportation as a means of transport, and have more electronic entertainment, labor-saving devices at home and more obesity rates [10, 14,]. Cultural aspects may also explain some patterns, through behavioral preferences. As in most areas of public health, data from several nations recommends that policies will be a vital part of combating ST and excess weight [4, 36, 42].

The relationship between ST with excess weight has consequences for future sedentary surveillance exploration and may inform the development of public health strategies and interventions to reduce ST in Latin America. To date, few studies have been performed on the association between ST and clinical health outcomes in Latin America. Lessons can be learned from Latin America countries as ST may continue to rise as they develop, requiring comprehensive strategies for promoting an active lifestyle while decreasing time spent in ST among populations. Intervention programs should consider device-based sedentary time patterns to decrease ST among inhabitants from Latin America [13].

The main strength of this study was the large sample size from eight Latin American countries using a standardized methodology across a consortium of participating countries. On the other hand, some limitations should be considered. Only urban areas were included in the study; thus, the samples are not nationally representative. Further, the cross-sectional design precludes determination of causality. The findings of this study show that ST increases the excess weight determined by NC in adults in Latin America. Excessive ST also increased the excess weight based on BMI, but not by WC. NC measurements were lower in the groups with less time sitting. More studies are needed to understand why NC is more sensitive to the risks of ST. In addition, there is a need to establish cut-points for ST that increase or decrease the excess weight.

## Conclusion

The present study concluded that categories of longer ST were associated with greater odds of excess weight. The excess weight caused by ST can be seen when using BMI and NC, but not by WC as excess weight indicators. This shows that excess weight indicators can respond in different ways to risk behaviors. Future studies should be carried out to estimate an acceptable value of ST in excess weight prevention.

## Data Availability

The datasets generated and/or analyzed during the current study are not publicly available due the terms of consent/assent to which the participants agreed but are available from the corresponding author on reasonable request. Please contact the corresponding author to discuss availability of data and materials.
